# Comparative mid-term outcomes of hemiarthroplasty and internal fixation in treating senile femoral intertrochanteric fractures: a retrospective cohort study

**DOI:** 10.3389/fmed.2026.1793952

**Published:** 2026-03-27

**Authors:** Wei Zhang, Yuhang Zhang, Haizhao Wu, Lingjian Tao, Xinhuan Lei, Zhongyi Chen, Jiajing Ye

**Affiliations:** Department of Orthopedics (Joint Surgery), Taizhou Hospital of Zhejiang Province Affiliated to Wenzhou Medical University, Linhai, Zhejiang, China

**Keywords:** femoral intertrochanteric fracture, hemiarthroplasty, hip, internal fixation, mortality

## Abstract

**Objective:**

This study aimed to compare the medium-term efficacy of hemiarthroplasty (HA) versus internal fixation (IF) in treating senile femoral intertrochanteric fractures and analyze their postoperative mortality and risk factors.

**Methods:**

A retrospective analysis was conducted on 192 elderly patients with unilateral femoral intertrochanteric fractures. Among these, 117 patients received IF, while 75 underwent HA. Comparisons were made between the two groups regarding operation time, hospital stay, blood transfusion rate, incidence of postoperative complications, American Society of Anesthesiologists (ASA) grade, number of concomitant medical diseases, Harris hip score post-operation, postoperative mortality, and survival curve of follow-up endpoints. Univariate analysis and the Cox proportional hazards model were applied to identify risk factors for postoperative mortality.

**Results:**

The HA group exhibited significantly lower incidence of postoperative complication and mortality rates compared to the IF group. At one-year post-operation, the HA group demonstrated superior Harris Hip Scores and better overall survival. Surgical method, ASA score, number of concomitant medical diseases, and postoperative blood transfusion rate were identified as significant factors influencing postoperative mortality in elderly patients with femoral intertrochanteric fractures. Furthermore, multivariate analysis confirmed that IF, a high ASA score, and the presence of multiple comorbidities were found to be independent risk factors for postoperative death.

**Conclusion:**

In this retrospective cohort, hemiarthroplasty was associated with lower postoperative mortality, fewer complications, and better early functional recovery compared with internal fixation. These findings highlight the importance of considering surgical method, comorbidities, and ASA score as key factors in improving postoperative prognosis.

## Introduction

Femoral intertrochanteric fractures are common clinical hip fractures in the elderly, accounting for approximately 8%–10% of all fractures (1). These fractures frequently occur in the setting of osteoporosis and are often comminuted. Elderly patients typically present with multiple comorbidities, some of which may be life-threatening. Studies have reported mortality rates as high as 27%–30% in elderly patients with femoral intertrochanteric fractures (2). Such patients are prone to complications such as accumulated pneumonia, lower extremity deep venous thrombosis, pressure ulcer, and urinary tract infection, which, in turn, reciprocally affect the primary injury (3). Identifying reasonable and effective treatment strategies that enable early ambulation while reducing postoperative complications and mortality has become a key focus of current research (4).

There are two surgical methods of femoral intertrochanteric fracture: internal fixation (IF) and hemiarthroplasty (HA). Although IF remains the standard treatment for femoral intertrochanteric fractures, the optimal therapeutic approach for elderly patients continues to be a subject of ongoing debate, with considerations of functional outcomes, complication rates, and long-term survival influencing the choice of treatment (5, 6). Due to the unique characteristics of elderly patients and the increasingly recognized biomechanical limitations of both extra- and intramedullary fixation (7), postoperative IF failure rates can reach as high as 20% (8, 9). Moreover, in unstable osteoporotic intertrochanteric fractures, achieving adequate reduction with IF is challenging, and early postoperative weight-bearing is often not possible. Prolonged bed rest significantly increases the risk of postoperative complications. In contrast, HA offers the advantages of early mobilization and faster recovery of joint function. Compared with IF, HA shows no statistically significant differences in hospitalization duration, pain control, transfusion rate, or mortality; however, the rate of implant-related complications is lower in patients undergoing arthroplasty. Joint arthroplasty is increasingly regarded as the best choice for femoral intertrochanteric fracture in the elderly (10, 11). The purpose of this study was to compare the mid-term outcomes of HA and IF in the treatment of femoral intertrochanteric fractures in elderly patients and to analyze postoperative mortality and associated risk factors.

## Materials and methods

**Figure 1:**
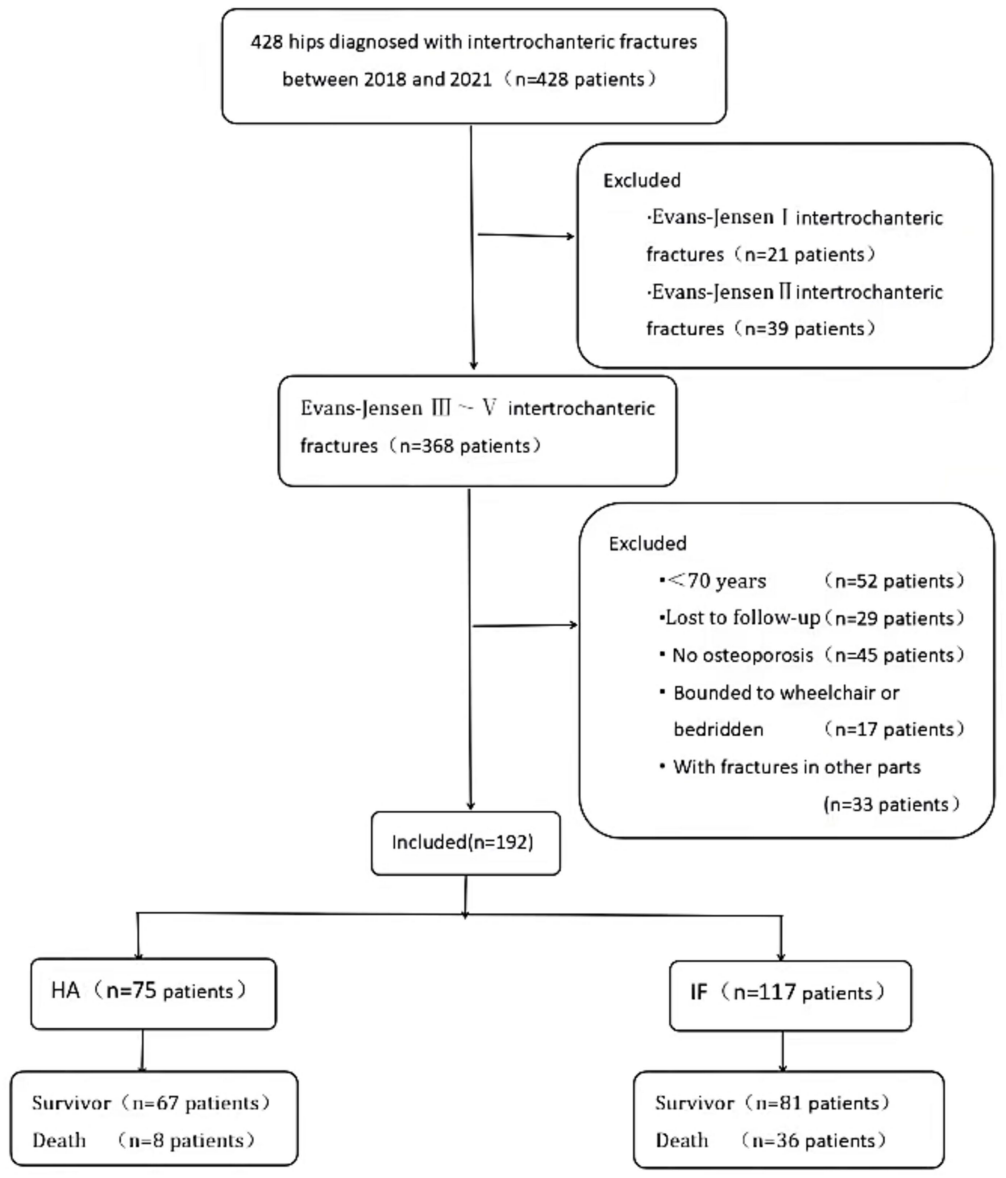
Flow chart demonstrating the patients’ exclusion criteria.

We conducted this study with approval from the institutional ethics committee. It included patients with intertrochanteric fractures treated surgically between 2018 and 2021. Inclusion criteria: ① unstable intertrochanteric fractures (Evans–Jensen III–V); ② patients aged 70 years or older; ③ osteoporosis (T score of ≤ − 2.5). Exclusion criteria: ① Evans–Jensen I or II intertrochanteric fractures; ② patients being fully wheelchair-dependent or bedridden before the fracture; ③ the presence of incomplete medical records; ④Patients lost to follow-up; ⑤ With fractures in other parts. The patient selection process is shown in Figure 1.

### Surgical procedures

All surgeries were performed by the same senior orthopedic team. The choice of surgical method was based on fracture stability, bone quality, comorbidities, and shared decision-making with patients’ families, following institutional guidelines.

**Figure 2:**
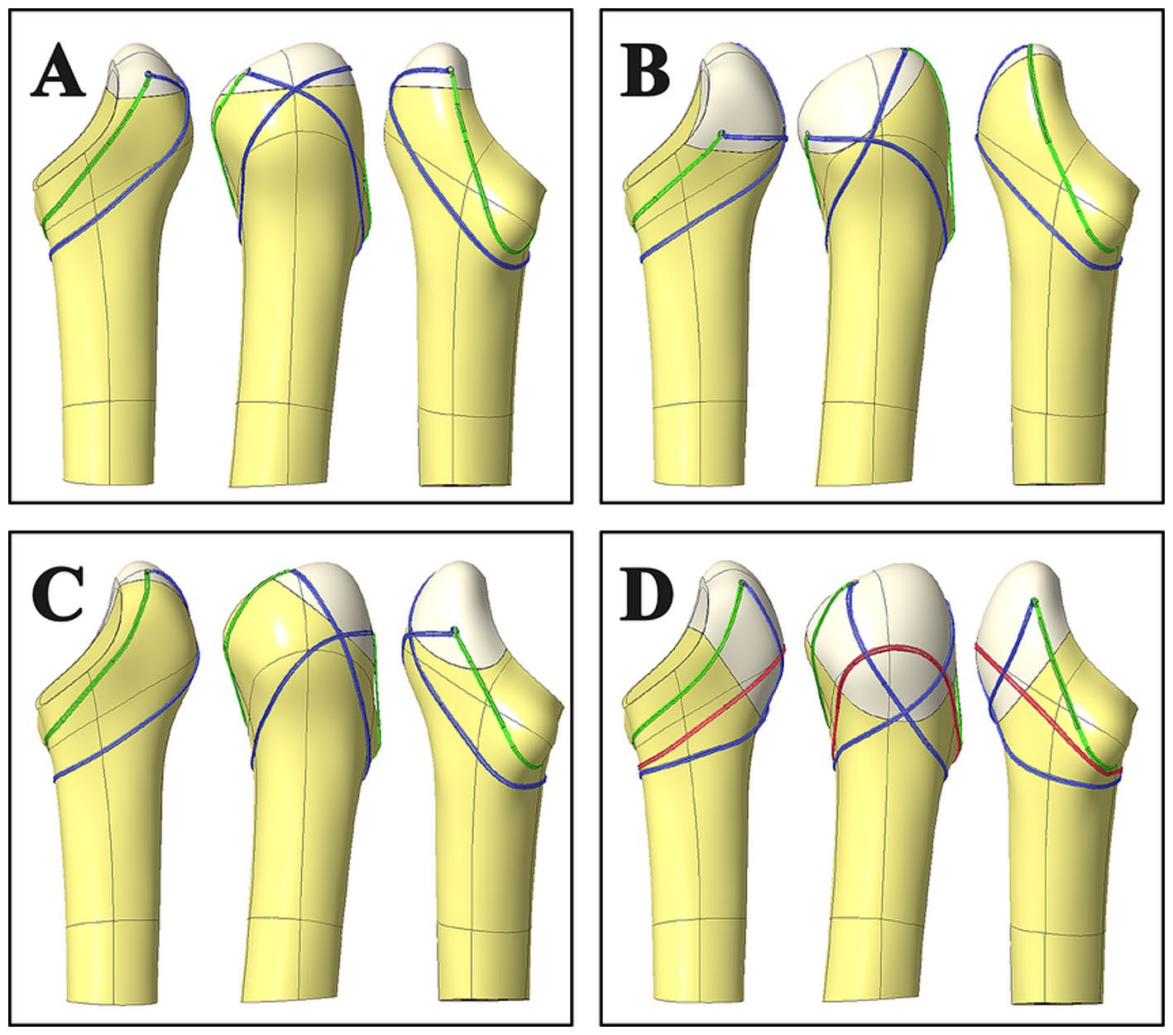
Wire winding methods for types **A**,**B**,**C**,**D** fractures, respectively. Type **A** fractures have transverse fracture lines from the tip of the greater trochanter to the level of the piriform fossa, fix with 8-shaped steel wire and circular steel wire. Type **B** fractures have oblique fracture lines from the tip of the greater trochanter to the base of the greater trochanter above the level of the lesser trochanter, the fractures run obliquely anteriorly and distally from behind the greater trochanter, fix with 8-shaped steel wire and circular steel wire, circular steel wire crosses in front of the greater trochanter of the femur. Type **C** fractures run obliquely posteriorly and distally from the upper anterior to the lower posterior, fix with 8-shaped steel wire and circular steel wire, circular steel wire crosses behind the greater trochanter of the femur. Type **D** fractures lines extend from the greater trochanter to the lower trochanter plane, fix with 8-shaped steel wire and two circular steel wires.

*HA group*: The patient was positioned on the contralateral side, with a subarachnoid block combined with epidural anaesthesia or general anaesthesia for pain management. A posterolateral approach was employed, with careful incisions through the skin, subcutaneous tissue, and fascia lata, followed by blunt dissection to expose the short external rotator group and the posterior edge of the gluteus medius muscle. The short external rotators were detached at their femoral attachment and retracted to protect the sciatic nerve. The upper femur was then exposed by subperiosteal muscle dissection, allowing assessment of the displacement of the intertrochanteric fracture. A conventional femoral neck osteotomy was performed to access the femoral head, and its diameter was measured. The greater trochanter fracture fragment was gently retracted forward, and the direction of the femoral medullary cavity was assessed. The medullary cavity was reamed to select an appropriately sized femoral stem prosthesis, which was then placed along the normal anteversion angle. After trial implantation of the femoral head model, the limb length and hip joint stability were carefully evaluated to ensure optimal positioning. Based on the displacement of the large and small trochanteric fragments, these fragments were mobilized and secured around the prosthesis using steel wires (Figure 2). Finally, the fascia lata was meticulously sutured, and the incision was closed, completing the procedure.

*IF group*: Surgery in the IF group was performed by means of Proximal Femoral Nail Anti-rotation (PFNA).

### Postoperative treatment

Antibiotics to manage infection, as well as anti-coagulation and pain-relief medication were administered routinely after the operation. Antibiotics should be used 30 min before surgery and stopped 1 day after surgery. Cefuroxime injection is commonly used, and vancomycin injection is used for allergies. Leukocyte and erythrocyte sedimentation rate and C-reactive protein levels were measured after the operation, and the healing of surgical incision was observed. Special treatment included the following: Patients receiving HA can use a walking aid to get out of bed and walk on the first day after surgery, and undergo hip, knee, and ankle joint functional exercises under the guidance of a rehabilitation therapist. Patients in the IF group should avoid weight-bearing on their affected limb for 1 month, and after 1 month, they can walk with partial weight-bearing assistance using crutches. After the operation, alendronate sodium and calcium tablets were administered to treat osteoporosis.

### Clinical measurements

The preoperative waiting time, operation duration, blood transfusion rate, hospital stay, incidence of postoperative complications, VAS scores, Harris score of hip function after operation, mortality within 1 year after operation, and survival curve of follow-up endpoints were compared between the two groups.

### Statistical analysis

SPSS version 22.0 (IBM, Armonk, NY, USA) was used for statistical analysis. Data measured such as age, BMI, bleeding volume, operation duration, duration of hospital stay, preoperative waiting time, follow-up time, VAS score, and Harris score were expressed by the mean ± SD. T-test and chi-squared tests were used for pairwise comparisons of numerical and parametric data, respectively. Survival curves were generated using the Kaplan–Meier method and compared by log-rank test. Univariate and multivariate Cox proportional hazards models were used to identify risk factors for 1-year mortality. The proportional hazards assumption was tested using log-minus-log plots and was satisfied. Reference categories were clearly defined (e.g., ASA < 3, IF, comorbidities <3). Statistical significance was set at *p* < 0.05 (two-tailed).

## Results

A total of 192 elderly patients with intertrochanteric fracture were enrolled in the study, with 75 patients in HA group, aged 83.14 ± 7.08 years (range, 70–100 years), a mean body mass index (BMI) of 21.46 ± 3.64 kg/m^2^ (range, 16.9–30.6 kg/m^2^), and 117 patients in the IF group, aged 81.84 ± 6.44 years (range, 70–97 years), a BMI of 21.62 ± 3.10 kg/m^2^ (range, 17.1–31.5 kg/m^2^) (Table 1). Table 1 demonstrates the list of preoperative comorbid systemic diseases noted in the patients of the current study, the median American Society of Anesthesiologists (ASA) score, and the Evans–Jensen type identified. The mean follow-up time was 34.16 ± 9.36 months (range, 12–50 months). Age, BMI, ASA score (of <3 vs. of ≥3), Evans–Jensen type III–V, and the number of comorbidities were not significantly different between the HA group and the IF group (*p* > 0.05) (Table 1).

**Table 1:** Comparison of patient demographics between the two treatment groups.

	HA (*n* = 75)	IF (*n* = 117)	*p* value
Age (years)*	83.14 ± 7.08 (70–100)	81.84 ± 6.44 (70–97)	0.271
Sex (female/male)	55/20	88/29	0.843
BMI (kg/m^2^)*	21.46 ± 3.64 (16.9–30.6)	21.62 ± 3.10 (17.1–31.5)	0.779
ASA score (<3/≥3)	38/37	55/62	0.722
Evans–Jensen III**	26 (34.6%)	40 (34.2%)	0.974
Evans–Jensen IV**	21 (28.0%)	26 (22.2%)	0.397
Evans–Jensen V**	28 (37.3%)	51 (43.6%)	0.585
Hypertension**	36 (48.0%)	61 (52.1%)	0.722
Chronic obstructive lung disease**	47 (62.7%)	60 (51.3%)	0.278
Diabetes mellitus**	10 (13.3%)	26 (22.2%)	0.378
Cerebrovascular disease**	38 (50.7%)	57 (48.7%)	0.857

IF, internal fixation; HA, hemiarthroplasty; ASA, score American Society of Anesthesiologists score; BMI, body mass index.

*Mean ± standard deviation (range).

**Patients’ ratio.

**Figure 3:**
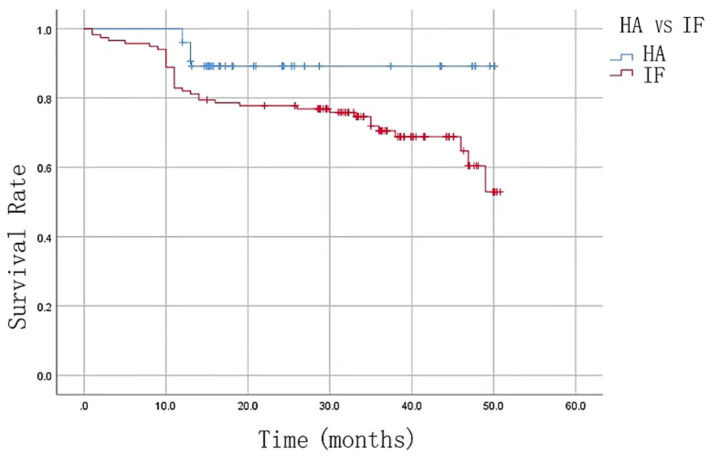
Kaplan–Meier survival curve at the follow-up endpoint.

**Figure 4:**
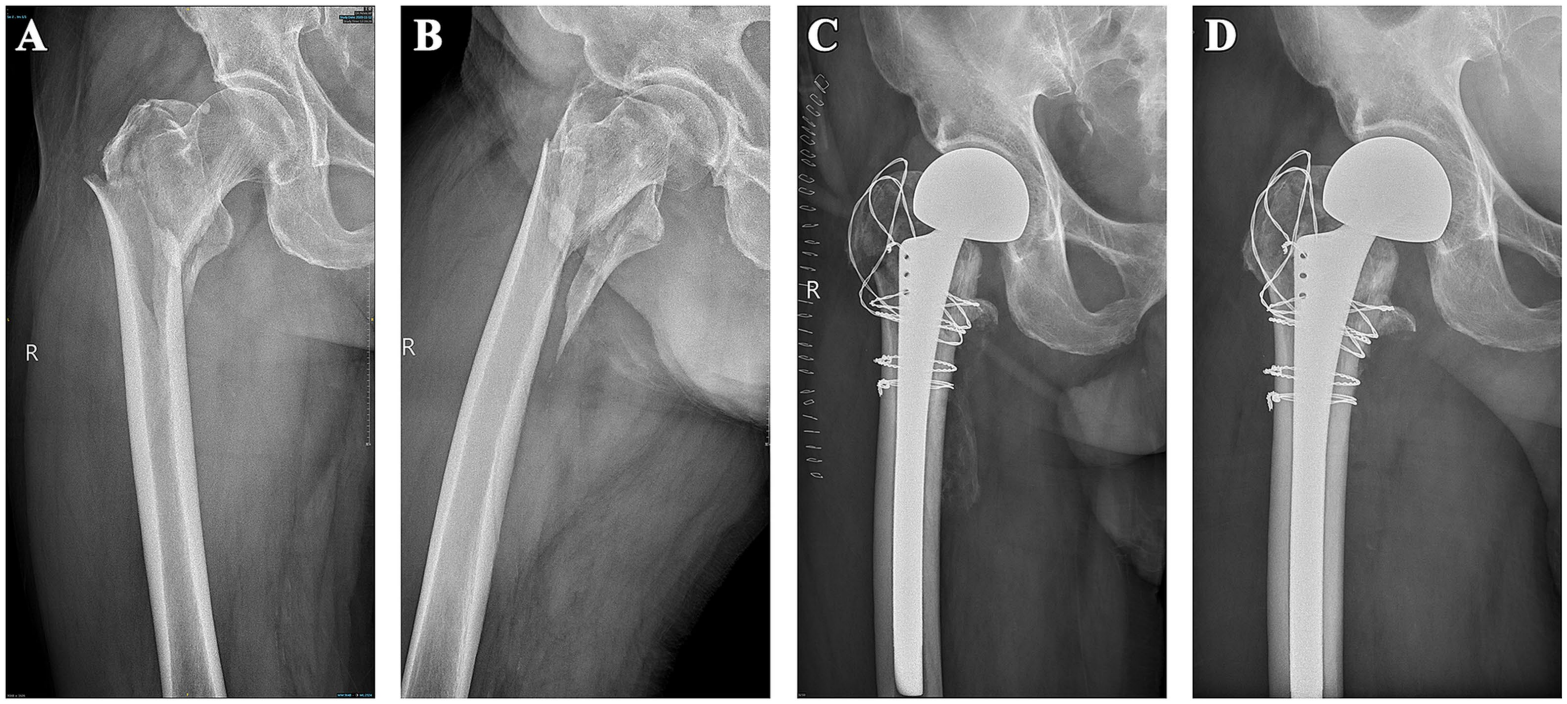
Characteristics of a typical patient’s case. **(A,B)** A preoperative frontal view of the lateral view of the upper and middle right femur reveal a comminuted fracture of the right femoral trochanter with obvious displacement. **(C)** X-ray on the first day after operation. **(D)** X-ray shows fracture healing and the position of the artificial joint is good 1 year after operation.

The HA group demonstrated a significantly longer operation time compared to the IF group (*p* < 0.05), but the HA group exhibited a lower incidence of complications (*p* < 0.05) and a significantly higher Harris hip score 1 week, 1 month, and 3 months after surgery (*p* < 0.05), highlighting superior functional recovery. Although VAS score, hospital stay and blood transfusion rates did not differ significantly between the groups (Table 2). The 1-year mortality rate was markedly lower in the HA group (10.7%, 8/75) compared to the IF group (30.8%, 36/117) (*p* < 0.05), reflecting better long-term survival outcomes. Consequently, the HA group showed a higher postoperative survival rate, reinforcing its potential as a more favorable treatment option for elderly patients with femoral intertrochanteric fractures (Figure 3). Typical cases are shown in Figure 4.

**Table 2:** Comparison of operative statistics between HA group and IF group.

	HA (*n* = 75)	IF (*n* = 117)	*p* value
Time from injury to surgery (days)*	3.23 ± 1.13 (2–7)	3.50 ± 1.67 (2–8)	0.248
Total hospitalization time (days)*	12.63 ± 3.80 (7–29)	14.34 ± 7.62 (5–30)	0.191
Operation time (minutes)*	74.85 ± 8.86 (65–95)	56.76 ± 10.90 (40–90)	**0.001**
Transfusion rate**	37.33% (28/75)	35.89% (42/117)	0.840
Postoperative complications**	10 (13.33%)	37 (31.62%)	**0.004**
Harris score (points)*			
1 week after operation	60.83 ± 3.83 (51–68)	51.84 ± 5.07 (42–65)	**0.001**
1 month after operation	68.97 ± 4.74 (58–82)	60.42 ± 5.95 (49–70)	**0.001**
3 months after operation	79.60 ± 7.54 (65–88)	70.34 ± 4.85 (62–82)	**0.001**
6 months after operation	85.77 ± 4.54 (75–93)	83.82 ± 5.14 (73–92)	0.099
1 year after operation	89.54 ± 3.89 (75–94)	88.87 ± 3.80 (71–93)	0.478
VAS (points)*			
1 week after operation	3.91 ± 0.95 (2–6)	4.34 ± 1.10 (2–7)	0.087
1 month after operation	3.00 ± 1.02 (1–5)	3.28 ± 0.98 (1–6)	0.240
3 months after operation	2.48 ± 1.01 (1–4)	2.65 ± 0.93 (1–5)	0.465
6 months after operation	1.77 ± 0.84 (0–3)	2.01 ± 0.80 (0–4)	0.250
1 year after operation	0.97 ± 0.65 (0–3)	0.98 ± 0.64 (0–4)	0.927

IF, internal fixation; HA, hemiarthroplasty. Bold values indicate statistical significance.

*Mean ± standard deviation (range).

**Patients’ ratio.

There was no significant correlation between operation time, time of hospital stay, incidence rate of postoperative complications, and mortality (*p* > 0.05). The Survivors group demonstrated a significantly lower transfusion rate compared to the Non-survivors group (*p* < 0.05) (Table 3). The latter four factors were included in a Cox regression analysis to determine the main risk factors for death. Using multivariate analysis, the simultaneous presence of ≥3 systemic diseases, the implementation of internal fixation surgery and an ASA grade of ≥3 were identified as the main risk factors leading to death (Table 4).

**Table 3:** Univariate analysis of risk factors for postoperative mortality in older patients with intertrochanteric fractures.

	Survivors (*n* = 148)	Non-survivors (*n* = 44)	*p* value
Age (years)*	81.46 ± 6.22	83.47 ± 7.14	0.058
Sex (female/male)	106/42	33/11	0.558
BMI (kg/m^2^)*	21.64 ± 3.05	21.99 ± 3.36	0.193
Surgical method (HA/IF)	23/125	8/36	**0.016**
ASA score (<3/≥3)	104/44	15/29	**0.000**
Time from injury to surgery (days)*	3.58 ± 1.55	4.05 ± 1.67	0.349
Total hospitalization duration (days)*	13.96 ± 7.59	14.48 ± 6.44	0.587
Operation time (minutes)*	59.62 ± 12.75	57.89 ± 10.96	0.280
Transfusion rate**	40.54% (60/148)	22.73% (10/44)	**0.031**
Postoperative complications**	25.00% (37/148)	22.73% (10/44)	0.758
Number of systemic comorbid diseases (<3/≥3)	122/26	30/14	**0.017**

IF, internal fixation; HA, bipolar hemiarthroplasty; ASA, American Society of Anesthesiologists; BMI, body mass index. Bold values indicate statistical significance.

*Mean ± standard deviation (range).

**Patients’ ratio.

**Table 4:** Multivariate analysis of risk factors for postoperative mortality in older patients with intertrochanteric fractures.

Variable	B	SE	Wald	*p*	HR	95% CI
ASA score (<3 vs. ≥3)	1.146	0.235	23.849	**0.000**	3.147	(1.98,4.986)
Transfusion (yes vs. no)	−0.316	0.222	2.014	0.156	0.729	(0.472,1.128)
Number of systemic comorbidities (<3 vs. ≥3)	1.066	0.229	21.759	**0.000**	2.904	(1.856,4.545)
Surgical methods [HA vs. IF]	1.647	0.545	9.143	**0.002**	5.193	(1.785,15.105)

IF, internal fixation; HA, hemiarthroplasty; ASA, American Society of Anesthesiologists; CI, confidence interval. Bold values indicate statistical significance.

## Discussion

Our study demonstrates that long-term hip function is superior in the HA group compared to the IF group. At 1 year post-surgery, Harris Hip Scores were significantly higher in the HA group than in the IF group. Patients undergoing HA are typically encouraged to ambulate on the first postoperative day, whereas those undergoing IF require a longer period before mobilization. The ability to engage in early functional rehabilitation is likely a key factor contributing to the better clinical outcomes observed in the HA group. This finding aligns with previous studies by Cobden et al. (12) and Chen et al. (13), which suggest that joint replacement combined with internal fixation provides better functional outcomes in the management of unstable intertrochanteric fractures in elderly patients. These results support the prioritization of HA for treating intertrochanteric fractures in this patient population. However, some studies have reported no significant difference in hip function between HA and IF, with shorter operation times and lower blood loss associated with IF. These findings suggest that HA should not be universally considered the first-line treatment for intertrochanteric fractures in the elderly (14, 15).

In terms of long-term survival, the HA group demonstrated a higher survival rate compared to the IF group, with a significantly lower incidence of postoperative complications. Specifically, the postoperative complication rate in the HA group was 13.3%, compared to 31.6% in the IF group. Despite greater surgical trauma and longer operation times in the HA group, there was no significant increase in blood transfusion rates, and the complication rate was notably lower than that in the IF group.

The reported 1-year mortality rates following surgery vary widely in the literature. Sidhu et al. reported a 17% mortality rate in patients over 70 years old treated with HA for unilateral intertrochanteric fractures (16). Similarly, Camurcu et al. (17) reported a 33.9% mortality rate in patients over 70 with unstable intertrochanteric fractures undergoing femoral head replacement with a cemented femoral prosthesis. In contrast, our study observed a 1-year mortality rate of 10.7% (8/75) in the HA group, compared to 30.8% (36/117) in the IF group (*p* < 0.05). Kaplan–Meier survival curve analysis further confirmed that patients in the HA group had a significantly better survival rate than those in the IF group.

Multivariate analysis in our study identified the choice of surgical approach as a significant predictor of postoperative mortality. The use of HA was associated with a marked improvement in postoperative survival, which may be attributed to the use of ventral compression tension band steel wire to secure greater trochanter fractures, providing a stable initial fixation. This stability may allow patients to mobilize earlier, facilitating functional rehabilitation. Early ambulation is crucial for elderly patients, as prolonged bed rest increases the incidence of complications and mortality postoperatively (18).

Age and preoperative comorbidities have been established as primary risk factors for postoperative mortality. As age increases and the number of comorbid conditions rises, the mortality rate following femoral intertrochanteric fractures also increases (16, 17). The aging process is often accompanied by declining organ function, reduced physical capacity, and diminished immune response, making older individuals more vulnerable to the stresses of fracture and surgery. Our results indicate that older patients with three or more pre-existing medical conditions prior to injury constitute a high-risk group for postoperative mortality. However, some studies argue that factors such as age, sex, and the number and type of comorbidities do not significantly impact mortality (19).

The ASA score is a widely used comorbidity index in clinical practice (20). Elcin et al. (21) found that an ASA grade of 4 was a major predictor of mortality after hip fracture in a study of 785 patients. Our univariate and multivariate analyses confirm that ASA grades of 3 or above are significantly associated with higher postoperative mortality.

The time from injury to surgery is another important factor influencing postoperative mortality after hip fractures (22). Many studies advocate for surgery to be performed as soon as possible, preferably within 48 h of admission, as this approach has been shown to reduce pain, complications, hospital stay duration, and postoperative mortality. Delayed surgery, on the other hand, is associated with higher mortality rates (23, 24). However, other reports suggest that the patient’s overall physical condition may play a crucial role in determining the timing of surgery. Patients with delayed surgery are often older and have more comorbidities, leading to poorer surgical tolerance and higher rates of complications and mortality (25). In our study, although the average time from injury to surgery was shorter in the HA group compared to the IF group, there was no significant difference in survival and mortality rates between the two groups in univariate survival analysis.

## Limitations

There are several limitations to this study. First, as a retrospective analysis, some clinical data were incomplete and could not be fully standardized. Second, the lack of randomization between the two patient groups introduces a degree of heterogeneity, which could act as a confounding factor, potentially affecting the long-term outcomes and introducing systematic bias. Therefore, prospective controlled trials with larger sample sizes are needed to more accurately compare the safety and long-term efficacy of the two treatment methods in elderly patients with femoral intertrochanteric fractures.

## Conclusion

Based on the results of this study, compared with internal fixation, elderly patients with femoral intertrochanteric fractures treated with hemiarthroplasty experience better recovery of joint function and overall functional capacity, along with higher survival rates. These findings may provide valuable guidance to treating surgeons in selecting the most appropriate surgical approach for elderly patients with femoral intertrochanteric fractures, potentially improving both clinical outcomes and long-term survival.

## Data Availability

The raw data supporting the conclusions of this article will be made available by the authors, without undue reservation.
